# Fear of Sexual Harassment and Its Impact on Safety Perceptions in Transit Environments: A Global Perspective

**DOI:** 10.1177/1077801221992874

**Published:** 2021-03-03

**Authors:** Vania Ceccato, Anastasia Loukaitou-Sideris

**Affiliations:** 1 KTH Royal Institute of Technology, Stockholm, Sweden; 2 University of California, Los Angeles, USA

**Keywords:** #MeToo! movement, fear of crime, sexual crime, public transportation, university students, gender

## Abstract

This study reports variation in safety perceptions in transit environments, based on samples of university students in 18 cities on six continents who responded to an identical 45-question survey (*N*= 13,323 university students). We explore potential links between students’ fears and sexual victimization and conclude that sexual harassment affects their behavior and mobility. Student mobility was affected by avoidance strategies prompting some transit riders to travel at particular times, on travel routes and settings that are deemed especially risky, or even avoiding using transit completely. Findings highlight the importance of city–country contexts for transit safety.

## Introduction

Sexual harassment and other forms of sexual violence in public spaces are everyday occurrences for women and girls around the world. The fear of sexual victimization may prevent women’s ability to participate in school, work, and public life (UN Women, 2017), limiting their life opportunities. This is particularly true in countries of the Global South where a large percentage of individuals, especially young women, are “transit captives,” having no access to private cars and relying on public transport for their travel needs (Yu & Smith, 2014).

Based on samples of university students in 18 cities around the world, this study examines how their concerns about sexual harassment in transit environments affect their perception of safety. We assess the variation of these students’ safety perceptions, examining how they differ because of their individual characteristics, transit mode, and city–country contexts. Finally, by comparing responses from students in different cities about their safety perceptions and their adoption of precautionary strategies, we seek to better understand whether fear affects these young riders’ mobility. This study is unique because it offers a comparative framework of analysis of the impact of sexual harassment on safety perceptions in transit environments in multiple geographic contexts, including countries in Asia, Europe, Africa, Australia, and North and South America, drawing from the experiences of students in cities of both the Global North and the Global South.

Many empirical studies indicate the occurrence of incidents of sexual harassment in transit environments (e.g., Ball & Wesson, 2017; Gardner et al., 2017; Halat et al., 2015; Hoor-Ul-Ain, 2020; Horii & Burgess, 2012; S. Lewis et al., 2020; Madan & Nalla, 2015; Natarajan et al., 2017; Neupane & Chesney-Lind, 2014; Orozco-Fontalvo et al., 2019; Tripathi et al., 2017). Sexual harassment is a multifaceted phenomenon that encompasses a variety of sexual behaviors. In this study, these include *nonverbal sexual violence/abuse*, such as stalking, exhibitionism, showing sexually explicit pictures, or making sexual gestures; *verbal sexual violence/abuse*, such as sexual comments, jeering or taunting, and asking questions about sexual activity; and *physical sexual violence/abuse*, which may involve behaviors such as touching, kissing, and more serious offenses such as rape. Although sexual harassment seems to be a global concern, the risk, fear, and impacts of victimization may vary highly from country to country (Gekoski et al., 2015). Knowing about students’ various degrees of victimization and fear helps to predict the impacts of this fear, from avoiding certain travel routes and/or times, to mobility impairment and isolation (Ceccato, 2017; Chowdhury & van Wee, 2020; James & Embrey, 2001; Loukaitou-Sideris & Fink, 2008; Pain, 1997; Paydar et al., 2017; Valentine, 1990). For young people in particular, such avoidance constitutes a barrier to movement, which in the long run may affect their life chances.

This article is composed of five sections. First, we discuss the literature on fear in transit environments. Then we frame the current study and research questions, and we follow with the results and discussion of our empirical study. The article ends with conclusions and recommendations for research and policy.

## Fear in Transit Environments: Brief Literature Review

Although public transport environments generate areas of convergence that are more prone to crime, victimization by any crime in these environments does not happen at random and may be influenced by a variety of factors. Similarly, how different travelers perceive risk or feel fearful in transit environments is not random but may vary because of their individual characteristics and the particular features and contexts of these environments. Fear, according to Warr (2000, p. 453), is “an emotion, a feeling of alarm or dread caused by awareness of expectation of danger.” Although understanding the factors that influence an individual’s fear of becoming a crime victim is important, it is also significant to consider the ways in which fear impacts an individual’s everyday life. Below, we discuss the concept of fear and its causes and impact on people’s lives.

### Causes of Poor Safety Perceptions

Individuals may feel unsafe for a variety of reasons, some of which may relate to the likelihood of being a victim of crime. Indeed, despite conflicting findings, international evidence indicates that previous victimization continues to be an important determinant of perceived safety (Hale, 1996; Hirtenlehner & Farrall, 2014; Otis, 2007; Yates & Ceccato, 2020). Witnessing other people’s victimization (particularly the victimization of a family member or friend) also affects an individual’s level of personal safety (for a review, see Skogan, 1987). Other individual factors may also determine the risk of victimization as well as safety perceptions. Scholars find that women, older adults, disadvantaged urban youth, members of ethnic minority groups, those who belong to the LGBTQI (lesbian, gay, bisexual, transgender, queer and intersex) community, those with disabilities, and those who are economically disadvantaged report higher levels of fear of crime (Alexander & Pain, 2012; Box et al., 1988; Garofalo & Laub, 1979; Iudici, 2015; Iudici et al., 2017; Pain & Smith, 2008; Sham et al., 2012; Yates & Ceccato, 2020).

Fear affects mobility and travel patterns (Loukaitou-Sideris, 2016). Feminist scholars have argued that “how people move (where, how fast, and how often) is demonstrably gendered and continues to reproduce gendered power hierarchies” (Cresswell & Uteng, 2008, p. 2). It is clear that gender distinctions in travel patterns exist in countries of the Global North and the Global South (Law, 1999; Tanzarn, 2008). Women typically make fewer job and business trips but more shopping trips and trips related to parental duties, elder care, and household obligations than men do. They also have more varied and complex activity patterns that often lead them to trip chain and higher overall numbers of trips. In many urban areas of the Global North and the Global South, women use public transportation more than men do (Cresswell & Uteng, 2008; Khan, 2013). Walking to/from a transit stop, waiting at bus stops or train platforms, and riding buses or trains expose women to the danger of sexual harassment and likely lead to higher levels of fear (Loukaitou-Sideris, 2005). The fear of harassment and crime in transit environments is probably one reason why women travel less frequently at night than men do and also avoid traveling during rush hours more than men do (Uteng & Cresswell, 2008).

Individual characteristics other than gender also influence fear and perceptions of safety. While young people are statistically more at risk of being victimized, older and/or disabled individuals tend to be more fearful (Furstenberg, 1971; Lagrange & Ferraro, 1989). Disability affects vulnerability to crime and may lead to high levels of fear (Iudici, 2015; Iudici et al., 2017). In Stockholm, for example, those who feel that they have one or more disabilities are twice as likely than the general population to report fear of being victims of assault or robbery (Ceccato, 2013), whereas in Brazil, scholars find that LGBTQI individuals, especially young women, tend to be overrepresented among those victimized by sexual harassment and sexual crime and declare to be more fearful while in transit (Nourani et al., 2020).

### Safety Perceptions in Transit Environments

Women typically report being more fearful than men in transit environments (Ceccato, 2013; Dymen & Ceccato, 2012; Loukaitou-Sideris, 1999; Uteng & Cresswell, 2008), despite the fact that men are more often victims of reported crime than women are (Morgan & Smith, 2006). On the contrary, sexual assaults and rapes, which primarily affect women, remain among the most underreported crimes (Solymosi et al., 2018). Koskela and Pain (2000) suggest that we create mental maps of feared environments and unsafe places based on our prior experiences as well as on media stories and the accounts of others, while Sandercock (2005) argues that expressions of fear of crime are actually fear of others. According to Yates and Ceccato (2020), fear is more prominent among specific groups of women, who have poor social contacts in their neighborhoods. Such fear ultimately leads to place avoidance strategies and other behavioral changes.

Certain environmental characteristics of places may generate fear. Studies have found that darkness, poor guardianship, desolation, lack of maintenance, physical and social disorder in a transit setting, and unkempt and abandoned buildings in its near vicinity may affect the safety perceptions of transit riders (Loukaitou-Sideris, 2009). Signs that nobody has control over the setting—e.g., litter, vandalism, and loitering—are thought to trigger fear of crime (Lewis & Maxfield, 1980) and are indicators of more serious crimes.

In addition, some potential dangers, located far beyond a particular setting, may cause fear and vulnerability and a sense of loss of personal security (Beck, 1992). Mass media coverage has an important role to play in this context, where the notion of “stimulus similarity” (e.g., when a reader of a newspaper or a social media site identifies themselves with a described victim who has been attacked or raped in a public transportation setting, generating the feeling, “it could have been me”) is suggested as important in explaining the reaction of fear (Winkel & Vrij, 1990).

### Temporal Patterns of Fear in Transit Environments

Fear and perceived risk can also vary temporally. Research evidence shows that safety perceptions may vary over the time of day, from weekdays to weekends, or during different seasons. For example, perceptions of safety may be more affected during peak hours (rush hours) of the day, when some crimes (e.g., pickpockets, jewelry snatching, groping) are facilitated by overcrowding (Block & Davis, 1999; Bradet & Normandeau, 1987; Ceccato & Uittenbogaard, 2014; Newton et al., 2014; Vanier & D’Arbois, 2018), or during off-peak hours, when crimes facilitated by the lack of natural surveillance (e.g., rape, aggravated assault) tend to happen (Austin & Buzawa, 1984; Transit Cooperative Research Program, 2001). Transit riders feel less safe in certain environments along the trip, particularly after dark (Smith & Cornish, 2012). Previous research indicates that fear intensifies after dark, most likely because more violent crimes happen during the evening/night at transportation nodes, when guardianship is poor and settings are empty (Yavuz & Welch, 2010). The gendered nature of women’s fear (Yates & Ceccato, 2020) is reflected in what has been called “the shadow of sexual assault” (Ferraro, 1996), making women pay higher costs than men for their “safety work” in public spaces, namely, the unnoticed work that goes into feeling safe (Vera-Gray, 2018).

### Fear and Its Impact

According to Gordon and Riger (1989), fear of crime leads to a sense that one should always be vigilant and alert. Such feelings have the power to modify and/or restrict people’s activities in everyday life (Jackson & Gray, 2010). With regard to impacts on mobility, Jackson and Gray (2010) distinguish between “functional” and “dysfunctional” fear. Functional fear leads to precautionary actions that may reduce both fear and risk of victimization, sometimes even prompting individuals to support activities that make crime and victimization more difficult to occur, such as participating in night patrols or neighborhood watch schemes (Gray et al., 2011). On the contrary, dysfunctional fear can paralyze individuals, leading to negative impacts, such as constrained mobility and avoidance of public spaces, in turn reducing life opportunities.

### Transit Safety in Different Contexts

The #MeToo! Movement^1^ has shown that sexual violence is an issue that transcends socioeconomic status. However, low-income women worldwide, who often are transit captives, may be more exposed to sexual harassment than any other group, and therefore are often found more fearful than those who have access to other transportation means. In the United States, for instance, research has found that low-income women are more fearful of crime in public spaces and transit environments than high-income women because they live in high-crime and unsafe neighborhoods (Loukaitou-Sideris, 2005). Various studies carried out in India, Bangladesh, Pakistan, and other southern hemisphere countries found that women are more dependent on public transportation and have less access to private transport than men (Hoor-Ul-Ain, 2020; Moreira & Ceccato, 2020; Peters, 2001; Sham et al., 2012; Yu & Smith, 2014). Even after controlling for the proportion of public transit users by gender, women are at greater risk of violence, sexual harassment, and sexual assault than men (Law, 1999; World Bank, 2015). Moreira and Ceccato (2020) also found that in Sao Paulo’s subway system, women are at higher risk of victimization than men, whereas men run higher risk of violence at end stations, both notably during late-night periods.

## Research Design

The empirical study examines the following research questions:

1. **Research Question 1:** Do safety perceptions vary because of individual characteristics of the student riders (such as gender, sexual orientation, previous sexual victimization) across all 18 cities?
2. **Research Question 2:** Do student transit riders declare feeling safer during the day than during the night?
3. **Research Question 3:** Are safety perceptions influenced by the particular city/country context?
4. **Research Question 4:** Does the transport mode (bus/train) affect expressed safety perceptions?
5. **Research Question 5:** To what extent, do feelings of lack of safety affect female students’ mobility, leading them, in particular, to exercise place/time avoidance strategies?

This study carried out an assessment of trends of sexual harassment among college students in 18 cities using an identical 45-question survey that inquired about the nature, type, settings, and extent of victimization in public transport settings and how it differs by gender, sexual orientation, and geographic context among college students in the different city/country contexts. The survey was translated into the local language of each city/country. Data collection took place between April and August 2018, with the exception of Lisbon, where researchers collected their data in early 2019. The cities included were: Vancouver, Canada; Los Angeles, CA, USA; San Jose, CA, USA; and Mexico City, Mexico in North America; São Paulo, Brazil; Rio Claro, Brazil; and Bogotá, Colombia in South America; Stockholm, Sweden; Huddinge, Sweden; London, UK; Paris, France; Lisbon, Portugal; and Milan, Italy in Europe; Tokyo, Japan; Guangzhou, China; and Manila, the Philippines in Asia; Lagos, Nigeria in Africa; and Melbourne in Australia. The appendix summarizes the characteristics of the samples by city, country, and continent.

For comparison purposes, we wished to include a variety of case studies from cities from different continents. Knowing that it would be difficult to access college students outside of our own countries, we utilized the user-list Transit Crime Research Network (https://maillist.sys.kth.se/mailman/listinfo/abe.kth.se_tcr-network) as well as our own network of contacts to identify scholars willing to translate our survey and distribute it to college students in their city. In this case, it was a convenience sample of cities, as we included researchers from this network who volunteered to take part in our study and distribute the survey to their students.

In most cities, we were able to reach and exceed a preset minimum sample of 300 students per sample; however, in two cities (Lagos and London), the samples were smaller, and these cities were excluded from some of the analyses. Overall, however, the average sample size was 650 students (ranging from a maximum sample size of 2,507 students in Lisbon, to a minimum sample size of 119 students in London). Some of the participating universities have a number of international students, but these students were underrepresented in the sample, not allowing for a comparison between domestic and international students. We have carried out an assessment of the methodology elsewhere; for more details, see the appendix and Ceccato and Loukaitou-Sideris (2020).

To create comparable data sets, we provided instructions to researchers administering the survey in each city, asking them to provide us with descriptive statistics from their survey data covering eight different themes. We have reported findings about these themes from the overall survey elsewhere (Ceccato & Loukaitou-Sideris, 2020). In this article, we wish to delve deeper and discuss one of these themes, namely, the students’ perceptions of safety in transit environments and the impacts that these perceptions have on their travel behavior. We asked students about their feelings of safety when traveling during the day and at night by bus and by train, while waiting at bus stops and station platforms, and while walking to/from the transit stop. Students had to respond to these questions on a Likert-type scale (from 1 to 5, with 1 = *never*, 2 = *rarely*, 3 = *sometimes*, 4 = *often*, and 5 = *always*). We report these findings in the next section.

## Results

### Safety Perceptions by Individual Characteristics

We sought to examine how individual characteristics such as gender, sexual orientation, and race/ethnicity may have impacted feelings of safety in transit environments. As we explain below, we were able to do a robust analysis in terms of gender, but our exploration of the effects of the other two variables faced challenges and was limited.

**Figure 1.**
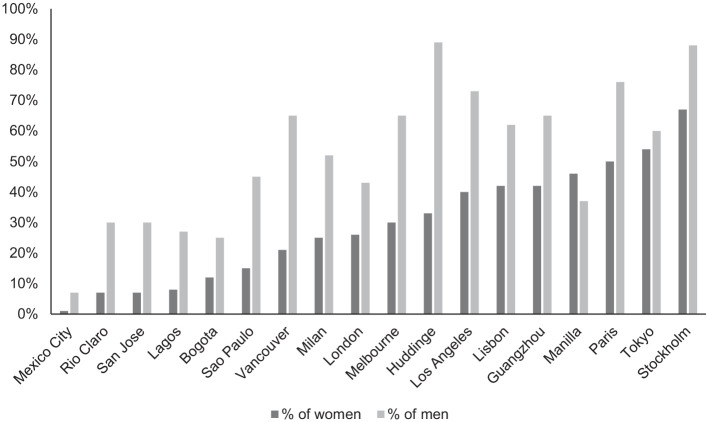
Percentage of students feeling “always” or “often” safe after dark on the bus in 18 cities, by gender. *Source*. Authors. *Note. N* = 18 cities, 13,323 university students.

1. *Gender.* Figure 1 shows that there were significant differences among the 18 cities in the percentages of students who reported feeling “always” or “often” safe in transit settings. For bus systems, Stockholm was found to be the safest, in terms of student perceptions, whereas Mexico City was perceived as the least safe by both male and female students in the respective samples. Overall, the importance of gender on fear was clear, as female students in all cities felt more unsafe in transit settings than their male counterparts, as Figure 1 shows. These perceptions of lack of safety were, as expected, more intense during the nighttime, but still there were significant variations among cities. Thus, while only 1% of female students and 7% of male students in Mexico City indicated feeling “always” or “often” safe in the bus system after dark, 67% of female students and 88% of male students in Stockholm felt safe in the buses and bus stops of their city after dark.
2. *Sexual orientation.* We were able to ask about sexual orientation in only 13 cities because in some cities questions about sexual orientation were not allowed by law or cultural norms. In eight of these 13 cities, LGBTQI students were victimized at somewhat higher rates than straight students. In Manila, Sao Paulo, and Rio Claro, somewhat higher percentages of LGBTQI students expressed feelings of lack of safety in transit settings than non-LGBTQI students. In Los Angeles, we did not find significant differences between these groups; however, when we intersected gender, we found significantly higher percentages of lesbian than gay students expressing feelings of fear in transit settings. Overall, however, the small number of LGBTQI students in the samples from some cities did not allow for robust results and comparisons between this group and other students. More future research is necessary on this topic and also on how differences among LGBTQI students (e.g., gay men vs. lesbian women) may affect feelings of safety.
3. *Race/ethnicity*. We were able to examine the impacts of race/ethnicity in only two cities (Sao Paulo and Los Angeles). The reason was that in many cities (e.g., Tokyo, Guangzhou, Manila, Milan), the vast majority of respondents shared the same racial/ethnic identities. In some other cities (e.g., Paris, Stockholm, Huddinge, Lisbon), the law does not allow questions about race/ethnicity, so we were unable to include such questions in the survey. Nevertheless, in Sao Paulo and Los Angeles, we found higher percentages of non-White students expressing fear in transit settings than White students did. For example, in Sao Paulo, more Black, mixed race, and indigenous women than White women reported never feeling safe during the day on the bus (14% vs. 9%), at the bus stop (33% vs. 26%), or on the train (11% vs. 5%). Similarly, in Los Angeles, White students (male and female) were more likely than non-White students to report “always” or “often” feeling safe in transit settings.

### Temporal Patterns of Safety Perceptions in Transit

As expected, students reported feeling safer during the day than at night. This is consistent with previous research that indicates that fear intensifies after dark, likely because more violent crimes happen during the evening/night in transportation settings because of desolation and low levels of guardianship. Figure 1 shows the percentage of respondents stating that they feel (always or often) safe after dark in all cities.

### Safety Perceptions by City–Country Contexts Across All 18 Cities

**Figure 2.**
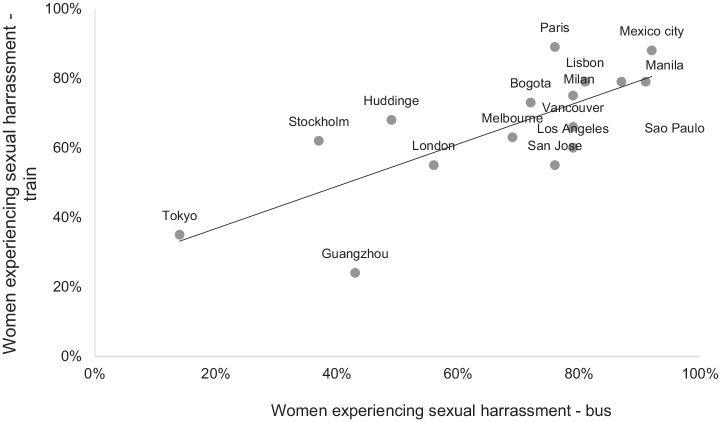
Women’s experiences with sexual harassment in bus- and rail-bound transit in selected cities (%). *Source*. Authors. *Note. N* = 16 cities. Rio Claro and Lagos were not included because the first does not have trains and the second has a metro system under construction.

Figure 2 shows two important findings: (a) respondents’ levels of victimization from sexual harassment vary highly by city, ranging from more than 70% in Sao Paulo and Lagos to around 30% in Guangzhou or Tokyo (for rail-bound transit), and (b) cities in Latin America and Africa top the scale of victimization.

By comparing Figures 1 and 2, we notice that those students from city samples characterized by higher levels of sexual harassment, such as Mexico City, Sao Paulo, or Lagos, also reported, ceteris paribus, feeling less safe. These findings might be related to the types of transit environments to which these students are exposed. We found, for example, that students who complained about problematic aspects of the physical or social environment of a setting (such as lack of lighting, desolate environment, presence of drunk people) at bus stops, on station platforms, or in transit vehicles were also those expressing higher levels of fear.

### Transport Mode (Bus/Train) Impact on Reported Safety Perceptions

**Figure 3.**
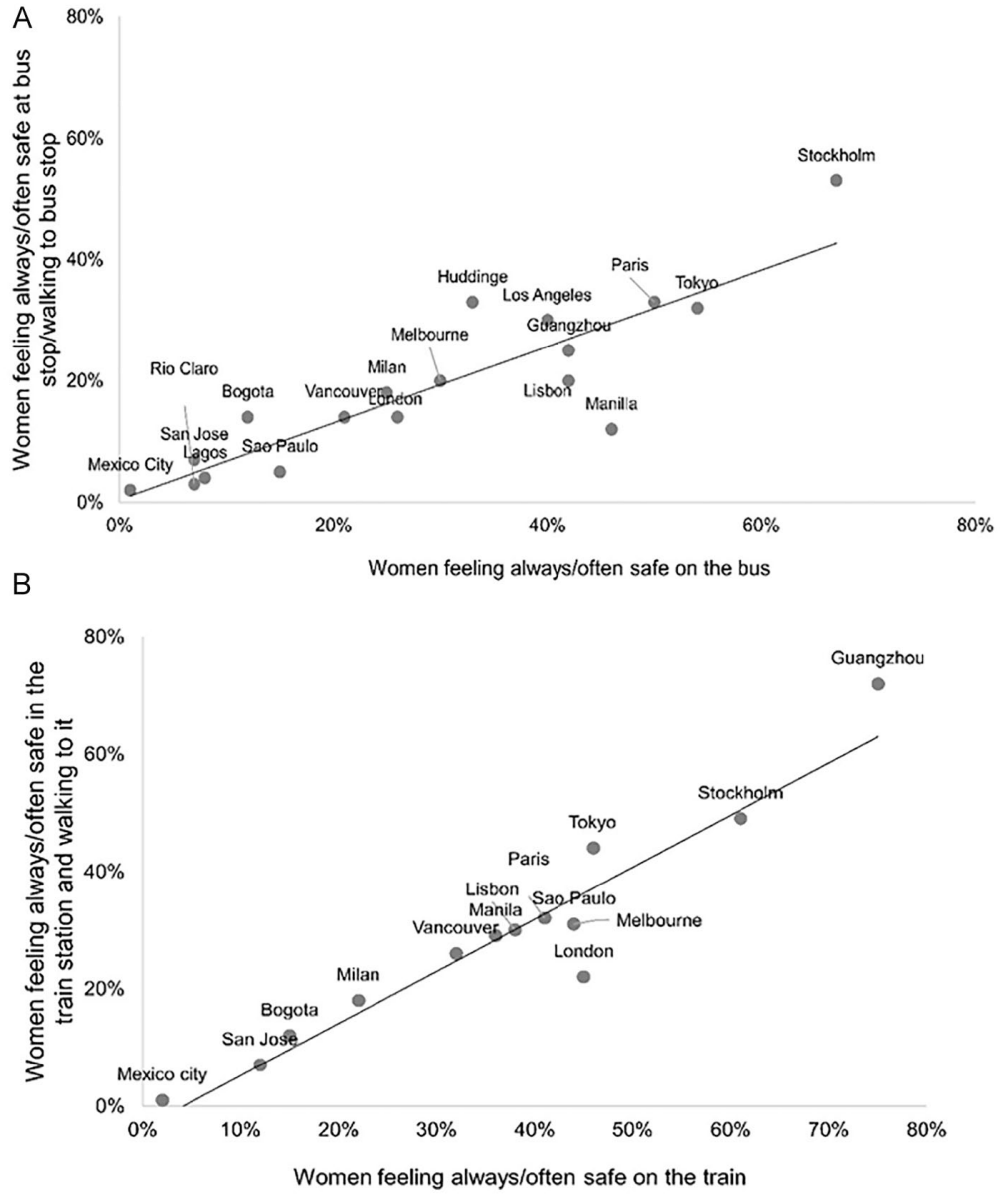
(A) Percentage of women feeling “always” or “often” safe after dark on the bus and at bus stop/walking to the bus stop (*N*= 18 cities) and (B) percentage of women feeling “always” or “often” safe after dark on the train and at train station/walking to the train station (*N*= 14 cities). *Source*. Authors. *Note.* Rio Claro and Lagos were not included because the first does not have trains and the second has a metro system under construction; Los Angeles and Huddinge did not collect data for this question.

We also found differences in students’ safety perceptions by transit mode (bus or rail), but which mode was perceived as safer differed by city. Figure 3(A) shows the percentage of female students feeling “always” or “often” safe after dark on the bus and at the bus stop/walking to the bus stop, whereas Figure 3(B) shows the percentage of female students feeling “always” or “often” safe after dark on the train and at the train station/walking to the train station.

Figure 3 shows there is a clear positive relationship between how women evaluated their safety on these transport nodes and on the way to them. In other words, cities where high percentages of female students reported feeling safe in these transport nodes (on the bus or train) tended also to have high percentages of female students reporting high levels of safety on the way to the nodes. However, an important difference was that overall women respondents reported feeling always or often safe more at the train station or bus stop than on the way walking to/from them.

### The Impact of Poor Safety Perceptions on Women’s Behavior

**Figure 4.**
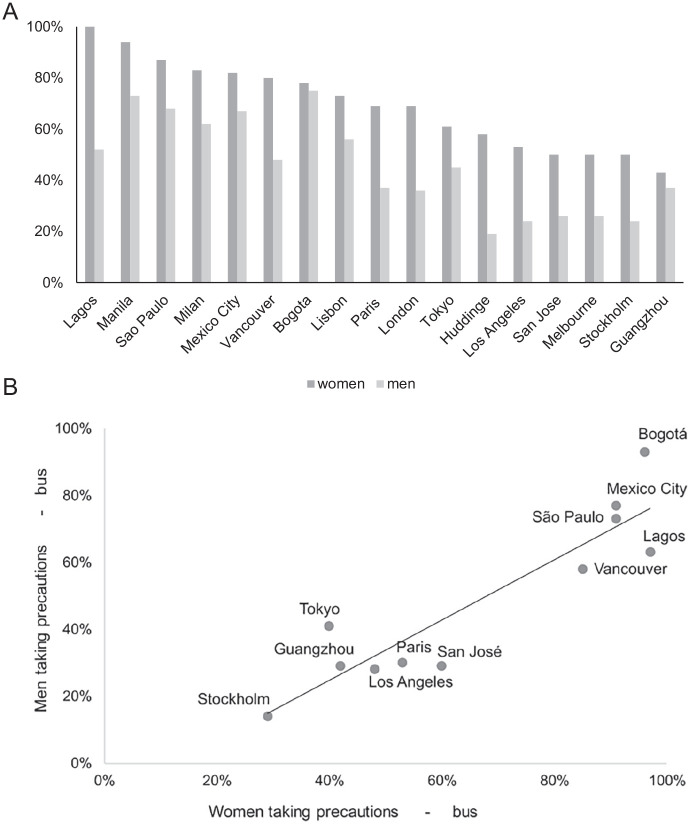
(A) Percentage of students taking precautions on the train system in selected cities and (B) precautionary measures taken on bus by women (*x*-axis) and men (*y*-axis) in selected cities (%). *Source.* Authors.

Sexual harassment in transit settings leads to the adoption of certain precautionary behaviors on the part of transit riders. Avoidance strategies prompt transit riders to travel only at particular times and to avoid travel routes and settings deemed as particularly risky, or even to avoid using transit completely. Figure 4(A) and 4(B) show that in all cities, female students took more precautions than male students did in all transit environments. However, the percentage of students taking precautions was context-specific and varied considerably by city. Figure 4(B) shows that for buses, both male and female students took precautionary measures that were proportionate to the levels of expressed perceptions of lack of safety. For instance, in Mexico City and Sao Paulo, where crime victimization is high, both male and female students reported taking precautionary measures more often than those living in other cities. This pattern was clearer for buses than for trains.

In addition, the types of common precautions that students took were sometimes similar and at other times different from city to city. For example, dressing only in a certain way to avoid sexual harassment was a very common strategy among many female students. In some cities, such as Tokyo and Guangzhou, more than half of the female riders reported following this strategy. This shows that globally, women often dress consciously to avoid attracting sexual attention and looks from men. Another example of a precautionary strategy, which was not that common among students in other cities, was weapon-carrying in Los Angeles, where one fifth of the female students reported carrying some kind of weapon (usually not a gun, but rather pepper spray, keys, or another sharp object) as a precaution when traveling.

During bus journeys, female students commonly reported seeking seats near the bus driver and also choosing bus stops to wait for the bus that were adequately lit. A common comment was that students felt the need to be vigilant and prepared when traveling, while many indicated that they stand ready to use a mobile phone to call for help if something happens. The survey indicated that men and women prepared themselves differently in situations they interpreted as risky. Some female students followed risk management strategies that prompted them to adopt reactive measures, such as dressing in a particular way, carrying some form of weapon, or using a backpack as a shield, as a means of preventing or minimizing the risk of exposure to harassment. For instance, in Stockholm, male students indicated that they often are prepared for theft or acts of violence in transit, whereas female students were often afraid of sexual attacks. While men usually want to show that they can defend themselves, women want to make sure they are noticed by bystanders in those environments (Ceccato et al., 2019), if a crime happens.

## Discussion

The findings discussed in the previous section help us respond to this study’s research questions. For one, this comparative, exploratory study showed definitively that sexual harassment is a common occurrence in the transit settings of different cities around the world. The large number of cities (18) and the diversity of continents from which data were collected give us a reasonable assurance that this is a global phenomenon. We found, however, that victimization rates from sexual harassment on transit vary significantly by city, with cities in certain regions (e.g., Mexico City, Sao Paulo, and Rio Claro in Central and South America, and Lagos in Africa) displaying significantly higher numbers of victimization among respondents than cities in other regions (e.g., Guangzhou and Tokyo in Southeast Asia). As expected, feelings of safety corresponded to rates of victimization, with students in cities with high victimization also having higher levels of fear. Yet, despite a clear link between victimization from sexual harassment and safety perceptions across all samples, a word of caution is needed here because previous studies show ambiguous links between victimization and fear of crime (Cates et al., 2003; Garofalo & Laub, 1979), indicating that its effect on people’s behavior and attitudes may be moderated by other factors. As already mentioned, experiencing or witnessing the victimization of others may also affect an individual’s perception of safety (Skogan, 1987), which can be the case in highly criminogenic contexts.

However, it was not only the city/country context that affected the responding students’ levels of fear. We found that students displaying certain individual characteristics were more likely to be fearful traveling by transit than others. Clearly, gender was an overriding determinant of fear, with female students in all cities expressing higher levels of fear and concern in transit environments than male students. Our limited analysis in some cities of the impact of race/ethnicity and sexual orientation on fear showed that being non-White and/or LGBTQI augmented feelings of fear and lack of safety. As the vast majority of the respondents in all cities were between the ages of 18 and 29 years, we did not examine the impact of age on fear. Despite such limitations, our findings corroborate other research evidence that indicates that women’s age alone is a strong predictor of sexual victimization (Madan & Nalla, 2015; Whitzman, 2007). Thus, it is the intersection of some of the aforementioned individual characteristics that interact and influence women’s vulnerability to crime and fear of crime (Crenshaw, 1989), and this is a topic that deserves further research.

Second, and as expected, we found that particular temporal and spatial characteristics of transit settings had an impact on perceptions of fear. Although different crimes tend to happen more during different spatial and temporal conditions, students in all cities, even after controlling for gender, showed greater fear during the nighttime. This is the time when certain environmental characteristics, such as desolation, darkness, and lack of natural surveillance are common at transit settings, affecting not only victimization (Liggett et al., 2001; Loukaitou-Sideris, 1999) but also safety perceptions of travelers while in transit (Ceccato, 2013; Natarajan et al., 2017). These findings call for an approach to women’s safety that goes beyond transport nodes and focuses on a multi-temporal “whole journey” perspective, examining different transit environments, during different hours of the day, weekdays, and seasons. For instance, the number of hours of sunlight in the spring in some study contexts (e.g., Scandinavian cities) may have mediated the effect of poor artificial lighting in some of their transit settings; survey responses may have been different if collected in the darkness of the winter.

Third, depending on the city, responding students tended to fear one transit mode more than the other; however, with the exception of two cities, levels of fear in one mode were related to levels of fear in the other mode. This may constitute evidence that safety perceptions are affected by the whole journey and might be more constant than previously expected. The differential levels of fear by mode within the same city may relate to the types of areas (high- or low-crime) that particular transportation systems are serving and also to differential levels of policing and investment in security technologies that may characterize different transit systems within a city. Such variations in safety perceptions have also been found in previous studies (Loukaitou-Sideris et al., 2002). We also found that in all cities, students’ fear was proportionately lower within the controlled environment of the transit vehicle than at the relatively open and less controlled environment of the street leading to the transit stop or at the transit stop.

Fourth, certain characteristics of the social environment of transit settings frequently associated with “social disorder” seemed to correlate with increased levels of fear. In all cities, the primary social environment variable associated with fear was the presence of intoxicated individuals. But depending on the city, the presence of panhandlers and people exchanging or consuming drugs was also related to higher feelings of anxiety and fear among responding students Ceccato and Loukaitou-Sideris (2020). The mechanisms linking visible deterioration of a setting to reported poor safety perceptions can be associated with Wilson and Kelling’s (1982) “broken window syndrome,” which suggests that unrepaired damage to property encourages further vandalism and other types of crimes, and acts of vandalism and public disorder function as symbols of the extent to which an area is in decline. Signs of physical deterioration are also thought to be more important determinants of fear of crime than the actual crime itself (D. A. Lewis & Maxfield, 1980). In addition, transport nodes are often surrounded by criminogenic land uses, such as bars, restaurants, and stores with ATM machines (Ceccato et al., 2011), which may also lead to riders’ feelings of lack of safety.

Finally, we found that female students were much more likely to take precautions while traveling than male students did. This, of course, relates to the higher level of anxiety that female students are feeling while traveling than their male counterparts, and the higher levels of perceived vulnerability and risk of sexual victimization. Jonathan Jackson (2009) also found that females tend to worry more often than males partly because they feel less able to physically defend themselves and have lower perceived self-efficacy. With regard to the consequences of fear, women differ from men in the way they are affected. As suggested by Jackson and Gouseti (2014), for some people, worry about crime can be a problem-solving activity and provide a sense of agency, whereas for others it can be something that damages their well-being. Some of the precautions in which female students are engaging certainly affect their mobility when they lead them to abandon transit completely or during certain times (time and/or place avoidance). Other precautions, such as dressing in a certain way or carrying some form of a deterrent to possible attacks, may be perceived as a nuisance; nonetheless, they also put constraints on the behavior of female students. The psychological and/or situational mechanisms that lead to one or another behavior in the context of transit environments (in the bus/train car, at the bus stop/station, and during the walk to the transit stop) have to be further investigated in future research in different country contexts

## Conclusions and Ways Forward

This study showed that sexual harassment in transit environments disproportionately affected female university students in our sample, representing a threat to the safety of their travel. This global study along with other literature from different cities shows that sexual harassment certainly impacts the unobstructed mobility of women, leading them to experience stress and fear in settings of everyday life. Sexual harassment varies temporally and across transit systems and city–country contexts, affecting different types of travelers.

This study has two important theoretical contributions related to the study of fear and victimization in transit environments. First, results show that levels of sexual victimization in transit environments seem to be associated with declared levels of perceived safety in these settings, regardless of the city or country contexts. These findings corroborate more general criminological work on the fear of crime that has shown that the perception of the likelihood of victimization is strongly correlated with expressed levels of fear about the event occurring (Ferraro, 1995). In the same vein, it would also be informative to investigate travelers’ sense of the seriousness of the short- and long-term consequences of sexual harassment for victims as well as nonvictims, who are fearful they might fall victim to such offenses.

Second, despite the importance of situational differences, gender was a strong determinant of both victimization and perceived safety in transit environments. The impact of gender was consistent across all samples; however, it was unclear whether fear of crime involved other psychological mechanisms linking gender to other intersectional characteristics (such as race/ethnicity, sexual orientation, status/identity, or disability) and/or “othering” processes and “fear of others” embedded in stereotyping certain types of individuals, groups, and places.

In summary, this comparative, exploratory study gave us some good indications and a better understanding about the fear that some young riders worldwide may feel while undertaking an everyday activity: using transit in their cities. Our study has also prompted more questions than it has answered. Therefore, we conclude this article by emphasizing the need for further research and painting its broad contours.

For one, we need to dive deeper to better understand and measure, through the employment of scientific random samples and the utilization of advanced statistics, the relative importance of different variables on fear in transit settings. We also need to explore how the size of a city and the complexity of its transportation network may affect feelings of fear. We know that individual variables affect fear, but the literature is still lacking large intersectional studies that show how characteristics such as race/ethnicity, age, or sexual orientation interact with gender to increase or decrease levels of fear.

We also need to better understand how particular environmental characteristics of a transit setting affect particular types of criminal and harassment behavior. For example, as we have noted, we can enumerate three different categories of harassment behavior, but we need more work to explore the situational circumstances and types of settings that may attract or deter each type of harassment behavior. The same also applies to safety perceptions, as we need to better comprehend how individual factors interact with the transit environment to affect it. As Jackson and Gouseti (2014) state, one has to consider not just the probability of victimization but also the controllability of crime and its consequences for a given individual in a given context— in this case, along the trip.

All studies about sexual harassment unambiguously indicate that women are victimized at a much higher level than men (e.g., Gekoski et al., 2015; S. Lewis et al., 2020; Moreira & Ceccato, 2020; Orozco-Fontalvo et al., 2019). However, as we found in our study, there were a number of male students in all cities (ranging from 11% in Tokyo to 65% in Mexico City), who reported also having been exposed to harassment behaviors in transit. We need to further study this incidence of male harassment to understand the types of harassment behaviors that affect men compared with the behaviors that affect women.

It would also be important to examine in more depth the types of precautions against harassment in cities—how they differ by gender, transit setting, and cultural norms—and to what extent they lead to positive and empowering behaviors (less fear) or to negative and paralyzing behaviors that constrain mobility (avoidance of travel because of fear). However, individual riders should not be left as the only ones responsible to protect themselves from sexual harassment and other criminal behaviors on transit. Therefore, it is also important to understand and evaluate best practices and lessons from transit systems around the world, which have proven to be effective in lessening harassment and crime.

## Appendix

**Appendix.** Summary of the Characteristics of 18 Case Samples of University Students.

City name	Country	Continent	Population	Universities involved	Sample size	Female/male
Tokyo	Japan	Asia	13,857,443	Multiple universities in Tokyo area	400	196/196
Guangzhou	China	Asia	14,904,400	SCUT and SCAU	402	202/191
Manila	The Philippines	Asia	12,877,253	University of the Philippines	316	166/144
Melbourne	Australia	Oceania	4,700,000	University of Melbourne (primarily)	517	350/151
Lagos	Nigeria	Africa	20,000,000 est.	University of Lagos	270	149/115
Bogotá	Colombia	South America	7,181,469	University of Los Andes	1065	531/522
São Paulo	Brazil	South America	12,176,866	USP (Cidade Universitária)	557	371/179
Rio Claro			201,473	UNESP	462	215/197
Los Angeles	USA	North America	3,792,621	UCLA	448	278/150
San Jose			1,046,079	San Jose State University	891	437/403
Vancouver	Canada	North America	631,486	Simon Fraser University	304	235/57
Mexico City	Mexico	Central America	8,855,000	CU Nezahualcóyotl	383	191/188
Stockholm	Sweden	Europe	965,232	KTH	1122	478/583
Huddinge			112,000	Södertörn University	309	230/64
Lisbon	Portugal	Europe	547,773	Multiple in Lisbon area	2507	1815/640
London	UK	Europe	8,170,000	Multiple in London area	119	69/47
Paris	France	Europe	2,140,526	Sciences Po	740	574/156
Milan	Italy	Europe	1,400,000	Università Cattolica del Sacro Cuore—Milan Campus	898	637/162

## Notes

1 #Me Too! is a movement against sexual assault and harassment that advocates for women who have survived sexual violence and harassment to speak out about their experiences. The movement spread quickly globally as a hashtag on social media after the sexual-abuse allegations against Hollywood film producer Harvey Weinstein in October 2017.
